# Effect of single-dose imipramine on chronic low-back and experimental pain. A randomized controlled trial

**DOI:** 10.1371/journal.pone.0195776

**Published:** 2018-05-09

**Authors:** Jürg Schliessbach, Andreas Siegenthaler, Lukas Bütikofer, Andreas Limacher, Peter Juni, Pascal H. Vuilleumier, Ulrike Stamer, Lars Arendt-Nielsen, Michele Curatolo

**Affiliations:** 1 Institute of Anesthesiology, University Hospital Zürich, Zürich, Switzerland; 2 Department of Anesthesiology and Pain Medicine, Inselspital, Bern University Hospital, Bern, Switzerland; 3 Chronic Pain Management, Lindenhof Hospital, Lindenhof Group, Bern, Switzerland; 4 CTU Bern, and Institute of Social and Preventive Medicine (ISPM), University of Bern, Bern, Switzerland; 5 Applied Health Research Centre (AHRC), Li Ka Shing Knowledge Institute of St. Michael’s Hospital, Department of Medicine, University of Toronto, Toronto, Canada; 6 Centre of Sensory Motor Interaction SMI, School of Medicine, University of Aalborg, Aalborg, Denmark; 7 Department of Anesthesiology and Pain Medicine, University of Washington, Seattle, WA, United States of America; Public Library of Science, UNITED KINGDOM

## Abstract

Antidepressants are frequently prescribed as co-analgesics in chronic pain. While their efficacy is well documented for neuropathic pain, the evidence is less clear in musculoskeletal pain conditions. The present study therefore evaluated the effect of the tricyclic antidepressant imipramine on chronic low-back pain in a randomized, double-blinded placebo-controlled design. To explore the mechanisms of action and the influence of drug metabolism, multimodal quantitative sensory tests (QST) and genotyping for cytochrome P450 2D6 (CYP2D6) were additionally performed. A single oral dose of imipramine 75 mg was compared to active placebo (tolterodine 1 mg) in 50 patients (32 females) with chronic non-specific low-back pain. Intensity of low-back pain was assessed on a 0–10 numeric rating scale at baseline and every 30 minutes after drug intake. Multimodal QST were performed at baseline and in hourly intervals for 2 hours. Pharmacogenetic influences of cytochrome P450 were addressed by CYP2D6 genotyping. No significant analgesic effect was detected neither on low-back pain nor on any of the sensory tests in the overall analyses. However, evidence for an interaction of the imipramine effect and CYP2D6 genotype was found for electrical and for pressure pain detection thresholds. Intermediate but not extensive metabolizers had a 1.20 times greater electrical pain threshold (95%-CI 1.10 to 1.31) and a 1.10 times greater pressure pain threshold (95%-CI 1.01 to 1.21) 60 minutes after imipramine than after placebo (p<0.001 and p = 0.034, respectively). The present study failed to demonstrate an immediate analgesic effect of imipramine on low-back pain. Anti-nociceptive effects as assessed by quantitative sensory tests may depend on CYP2D6 genotype, indicating that metabolizer status should be accounted for when future studies with tricyclic antidepressants are undertaken.

## Introduction

Tricyclic antidepressants (TCAs) are frequently used in chronic pain therapy. Albeit most effective in neuropathic pain [1,2], they are as well prescribed in many other painful conditions, including fibromyalgia, headaches or chronic low-back pain. There are several reasons for using TCAs in chronic low-back pain: they favorably influence concomitant mood disorders, may improve sleep quality and exert an analgesic effect. The rationale for an analgesic effect is the modulating action at serotonergic and noradrenergic pathways involved in nociceptive transmission and endogenous pain modulation [3].

Some clinical studies have reported beneficial effects of TCAs in chronic low-back pain [4,6], assessed by outcomes such as pain intensity or functional improvement after trial periods of several weeks. These findings, however, might as well be related to the effects of TCAs on sleep quality or mood, and therefore do not provide sufficient mechanistic information about the action of these drugs. On the other hand, most of the mechanistic knowledge about the anti-nociceptive properties of TCAs is derived from studies in animals or human volunteers rather than pain patients. Here, it has indeed been shown that TCAs exert acute anti-nociceptive effects [7–11] as assessed by quantitative sensory tests (QST), already after a single TCA dose. With regard to acute pain, there is currently hardly any evidence for the use of antidepressants [12].

Pain practitioners are thus faced with two questions: does clinical improvement really suggest a genuine analgesic effect of a TCA, and does anti-nociception assessed by QST also imply a clinically meaningful treatment outcome?

An answer to this question might be provided by combining both the mechanistic and the clinical approach in the same study. Unfortunately, studies comparing the analgesic effect of TCAs in chronic pain with anti-nociceptive effects using QST are sparse. The present study was part of a larger project investigating the prediction of the effect of different drugs on chronic low-back pain [13] (S1 Appendix). Here, we report the results of a randomized, double-blinded and placebo-controlled sub-study which investigated the effect of a single oral dose of the TCA imipramine on both low-back and experimental pain in humans. Effects on low-back pain and experimental pain (QST) were analyzed separately for genetic polymorphisms of cytochrome P450 2D6 (CYP2D6), the major metabolic pathway of imipramine.

## Methods

### Setting

The study took place at the University Department of Anesthesiology and Pain Therapy, Inselspital Bern, Switzerland between July 2010 and April 2014. It was approved by the local ethics committee Kantonale Ethikkommission Bern (KEK 213/09) and registered at clinicaltrials.gov (NCT01179828). All participants gave written informed consent prior to inclusion.

### Patients

Consecutive patients aged between 18 and 80 years with chronic low-back pain of at least 3 months duration were recruited by advertisement in local newspapers and from our outpatient pain clinic. Exclusion criteria were pain intensity at rest <3/10 on the numerical rating scale (NRS) at the time of testing (whereby 0 = no pain and 10 = worst pain imaginable), suspected radicular pain (as defined by leg pain associated with an MRI finding of a herniated disc or foraminal stenosis), signs or suspicion of neurological dysfunction at the tested sites, pregnancy (as assessed by pregnancy test), breast feeding, ongoing treatment with an antidepressant, opioid or anticonvulsant, intake of centrally active substances (including drug or alcohol abuse), known allergy or pharmacological contraindications to imipramine or tolterodine (active placebo), multi-site or widespread pain, systemic inflammatory or rheumatological disease, and major depression (Beck Depression Inventory short form score >9). Analgesic medication had to be stopped one week before the first experiment. Only acetaminophen or ibuprofen were allowed as rescue medication until 24 hours before the experiments. Patients unable to stop their analgesic regimen were not recruited.

### Study medication

Previous studies in healthy volunteers used single oral imipramine doses of 100 mg [10,11] with no serious adverse effects. Nevertheless, given that the present patient population would be older and maybe more prone to side-effects, a single oral dose of imipramine 75 mg was chosen. The anti-cholinergic compound tolterodine 1 mg was used as active placebo in a cross-over fashion. Tolterodine is prescribed for hyperactive bladder disorders. It is a specific antagonist at muscarinic M2- and M3-receptors and should be devoid of any analgesic effects, but mimics some of the sedative side effects such as blurred vision, drowsiness and sleepiness. This allowed for better blinding of patients and investigators than an ineffective placebo. A minimal wash-out period of one week between sessions was ensured. The drugs were administered as identical-looking red gelatin capsules in random order and in a fasting state. Blinding and randomization were provided by the hospital pharmacy as described in detail in the study protocol [13] (S1 Appendix).

### Intensity of low-back pain

Intensity of low-back pain was assessed on a 0–10 NRS at baseline and in intervals of 30 minutes up to 2 hours after drug intake, both in the supine position and after 10 minutes of sitting. The patients’ global impression of change scale (PGIC) was assessed on a 7 point scale ranging from “1 = very much improved” over “4 = no change” to “7 = very much worse”, in intervals of 30 minutes, starting 30 minutes after drug administration.

### Quantitative sensory testing

Quantitative sensory testing was performed at baseline as well as one and two hours after drug administration. All tests were performed at the more painful body side. In case of bilateral or midline pain, the side was randomly selected. The detailed methodology is described elsewhere [13] (S1 Appendix). In brief, pressure pain detection and tolerance thresholds (PPDT and PPTT) were measured at the second toe using an electronic algometer (Somedic AB, Horby, Sweden). Electrical single pain threshold (ESPT) and electrical repeated pain threshold (ERPT with 5 stimuli at 2 Hz inducing temporal summation) were measured using a constant current stimulator (Digitimer Ltd, Welwyn Garden City, UK) and two surface electrodes attached below the lateral malleolus in the innervation area of the sural nerve. Heat pain detection and tolerance threshold (HPDT and HPTT) as well as cold pain detection threshold (CPDT) were measured at the ipsilateral leg at the L5-dermatome and the ipsilateral forearm at the C6-dermatome using a Peltier thermode (TSA II, Medoc, Ramat Yishai, Israel). Conditioned pain modulation (CPM) was assessed at baseline only using the cold pressor test of the contralateral hand as conditioning stimulus. Electrical train of five stimulation at an intensity 1.2 times the ERPT was rated by the subjects on a 0–10 NRS before and during the cold pressor test. The percent decrease in ERPT rating during the cold pressor test was calculated as an indication of CPM. Furthermore, the time until cold pressor pain reached 7/10 NRS was recorded. Hand immersion in ice water was repeated after 1 and 2 hours and the time to NRS 7 was noted. For all tests, triplicate measurements were recorded. A complete series of training measurements was performed before baseline assessments, in order to familiarize patients with the procedure.

### Descriptive variables and side effects of medication

The following descriptive variables were assessed on a questionnaire before the first study session: age, sex, body mass index (BMI), pain duration in years, history of surgery due to the painful condition, average pain intensity during the last 24 hours on a 0–10 NRS, pain-related life interference from the multidimensional pain inventory (MPI), catastrophizing scale and Beck Depression Inventory (BDI). Patients rated the intensity of nausea, dizziness and sedation every 30 minutes after drug intake on a 0–10 NRS.

### Genotyping

Genomic variants of cytochrome CYP2D6 were assessed by real time polymerase chain reaction (PCR) and identification of specific variants by means of melting curve analysis. The following alleles were examined: CYP2D6 *3, *4, *5, *6, *8, *10 and *41 as well as gene multiplication. Alleles with none of these variants were categorized as “wild-type” (wt, *1). Translation of the genotypes into a qualitative measure of phenotype was made according to Gaedigk’s system of “activity scores” [14]: alleles *3,*4,*5,*6,*7, and *8 were assigned a value of 0, alleles *10 and *41 a value of 0.5, the wild type (wt) allele a value of 1, and wtxN (representing multiplication of the wt allele) a value of 2. The sum of the values assigned to each single allele resulted in a CYP2D6 activity score. Activity scores of 0 correspond to poor metabolizers (PM), scores of 0.5–1 to intermediate metabolizers (IM); scores of 1.5–2 to extensive metabolizers (EM) and scores of 3 to ultra-rapid metabolizers (UM) with a duplication or multiplication of a functional allele.

### Statistical analyses

Normal distribution of variables was checked by visual inspection using quantile plots. Continuous and ordinal variables that were normally distributed (NRS) or normally distributed after log-transformation (PPDT, PPTT, ESPT, and ERPT) were analyzed by linear mixed models, with treatment group, time point and their interaction as covariates. The models were adjusted for baseline values and treatment phase (verum first vs. placebo first) in order to account for a possible learning effect. A carry-over effect was excluded by design (wash-out period between the phases) and was not tested for. A random intercept was added for each subject (to account for intra-subject correlation) and a random intercept and slope for each subject in each treatment phase (to account for repeated measures). Correlations between subsequent measurements were modeled with a first order autoregressive correlation structure. The treatment effect was calculated over all time points (joint p-value) and at each time point based on marginal models. HPDT and HPTT which were truncated at 50.5°C were analyzed by separate mixed tobit regression models at each time point with treatment group and phase as covariates and subject ID as panel variable. CPDT was dichotomized (reaching 0°C) and analyzed by separate logistic GEEs at each time point with treatment group and phase as covariates and subject ID as panel variable. Sample size considerations were based on a larger project and have already been published [13] (S1 Appendix). For a subgroup analysis, CYP2D6 metabolizer group and its interaction with treatment group were added to the models. All statistical analyses were made using STATA (STATA Corp, College Station, Texas).

## Results

**Fig 1:**
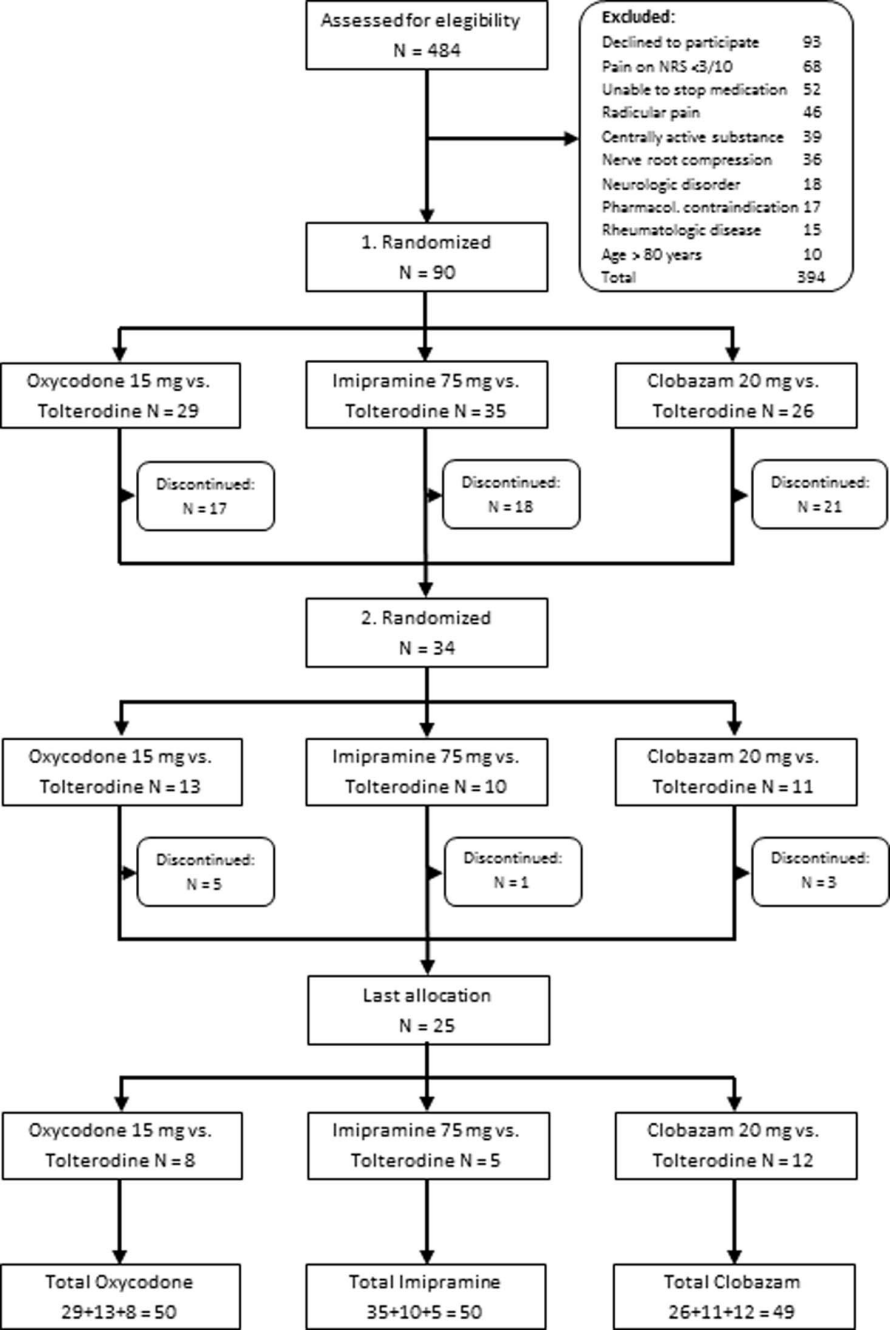
Patient flow-chart. Flow-chart displaying study design and treatment allocation.

A total of 50 patients were enrolled (32 females), with a mean age of 54.4 years (SD 17.3 years) and average pain duration of 11.2 years (SD 12.3). Descriptive variables are detailed in Table 1. Study design and treatment allocation are shown in Fig 1. The CONSORT checklist can be found in S2 Appendix.

**Table 1:** Descriptive variables.

Number of patients	50
Age (years)	54.4 ± 17.3
Sex (female)	32 (64%)
BMI (kg/m^2^)	26.6 ± 4.6
Pain duration (years)	11.2 ± 12.3
Previous back surgery (yes)	10 (20%)
Average pain in 24h (0–10 NRS)	5.6 ± 1.8
Impairment of daily life (min: 0, max: 10)	2.4 ± 1.2
Catastrophizing score (min: 1, max: 6)	1.6 ± 1.2
Beck Depression Index (min: 0, max: 21)	2.3 ± 2.3
CYP2D6 PM	3 (6%)
CYP2D6 IM	20 (40%)
CYP2D6 EM	26 (52%)
CYP2D6 UM	1 (2%)

Continuous data are presented as means and standard deviations, ordinal data as numbers and percentages. BMI = Body mass index, NRS = numeric rating scale, PM/IM/EM/UM = poor, intermediate, extensive and ultrarapid metabolizers of CYP2D6.

### Intensity of low-back pain

In terms of relief of low-back pain, imipramine was at no time point significantly different from placebo, neither in the sitting nor in the supine position. Pain intensity in supine position decreased from 4.2 to 2.6 after 2 hours in the imipramine arm and from 4.0 to 2.5 in the placebo arm (treatment effect 0.02 (-0.51 to 0.56), joint p = 0.950). The PGIC showed a corresponding trend to minimal improvement in both arms (imipramine 3.6 (3.3 to 3.9), placebo 3.4 (3.1 to 3.7), treatment effect -0.2 (-0.6 to 0.2), joint p = 0.669).

### Quantitative sensory testing

**Fig 2:**
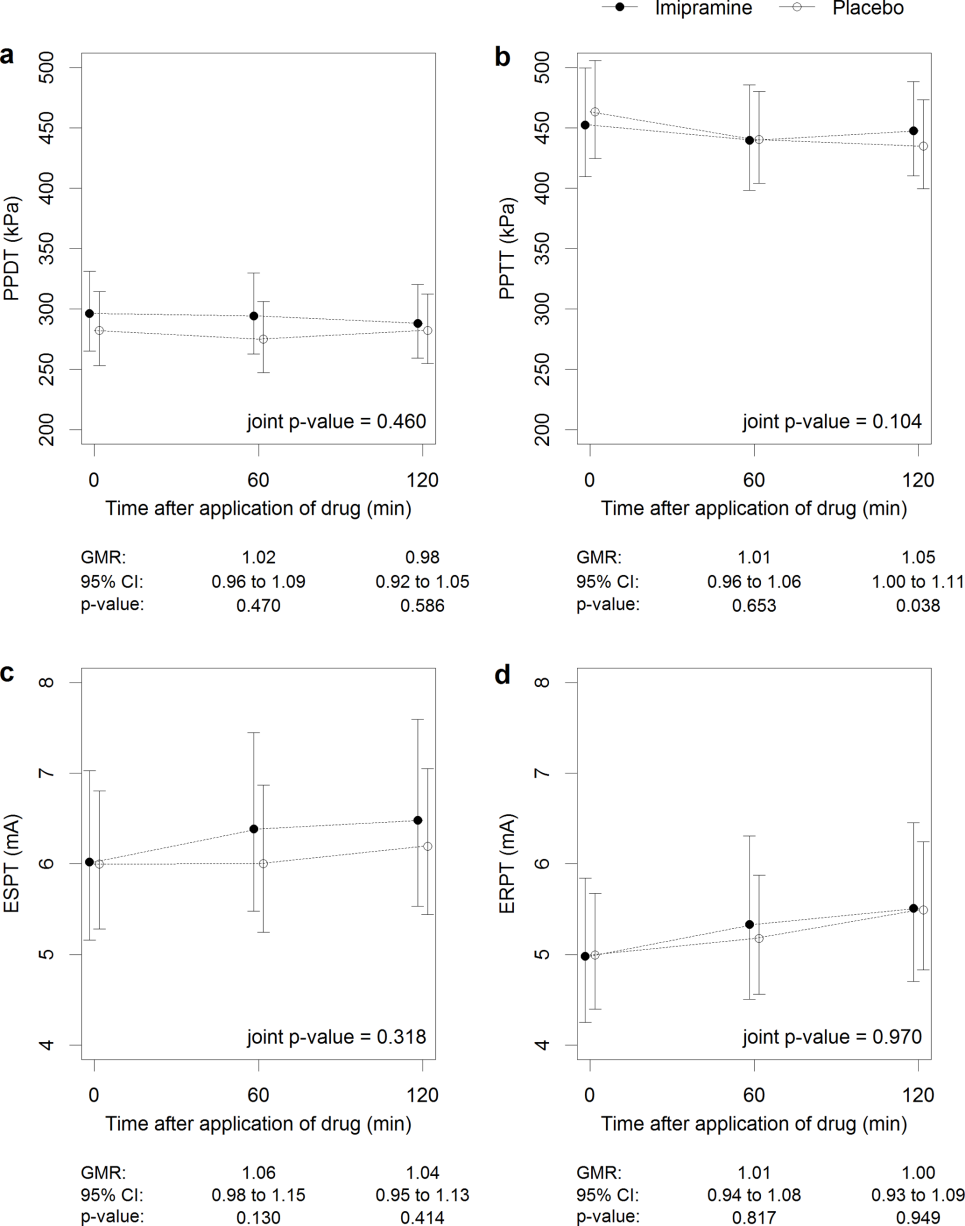
Effect of imipramine on quantitative sensory tests. Imipramine effect on pressure pain detection (PPDT, a) and tolerance thresholds (PPTT, b), and on electrical single pain detection threshold (ESPT, c) and electrical repeated pain threshold (ERPT, d) in all patients (n = 50), independent of genotype. GMR = geometric mean ratio.

No effect could be observed on the QST measures, with the exception of a 1.05 times higher PPTT in the imipramine session after 120 minutes (95%-CI 1.00 to 1.11), p = 0.038, Fig 2). The remaining QST parameters (i.e. ESPT, ERPT, PPDT, PPTT, time of ice immersion and all thermal pain tests) showed no significant change over time.

### Genotyping—Effect of imipramine by CYP2D6 metabolizer status

During data analysis, QST results were plotted for each CYP2D6 metabolizer group. It became apparent upon visual inspection that anti-nociceptive effects might depend on metabolizer status. Subgroup analyses were therefore performed in order to check for interactions of imipramine effect with metabolizer status. However, this subgroup analysis had to be restricted to intermediate (n = 20) and extensive metabolizers (n = 26, thus roughly comparable in subsample size), because there were too few poor (n = 3) and ultra-rapid (n = 1) metabolizers. All patients were successfully genotyped. The frequency of each of the four metabolizer phenotypes is displayed in Table 1. Mutant allele frequencies were 22.0% for *10, 19.0% for *4, 8.2% for *41, 5.0% for *5. Mutant alleles for *3, *6 and *8 were not found at all. These numbers are in line with Gaedigk’s in a Caucasian population [14].

Again, relief of low back pain after medication was not different between imipramine and placebo, but a significant effect on QST could be detected in intermediate metabolizers of CYP2D6. This was most pronounced for ESPT: intermediate metabolizers had 1.20 (1.10 to 1.31) and 1.15 (1.04 to 1.26) times higher ESPT 60 and 120 minutes after imipramine compared to placebo (p<0.001 and p = 0.006, respectively), whereas it remained almost unchanged in extensive metabolizers. This interaction was highly significant (p = 0.007). A similar but weaker trend for interaction could be observed in the ERPT measurements (interaction p = 0.079).

**Fig 3:**
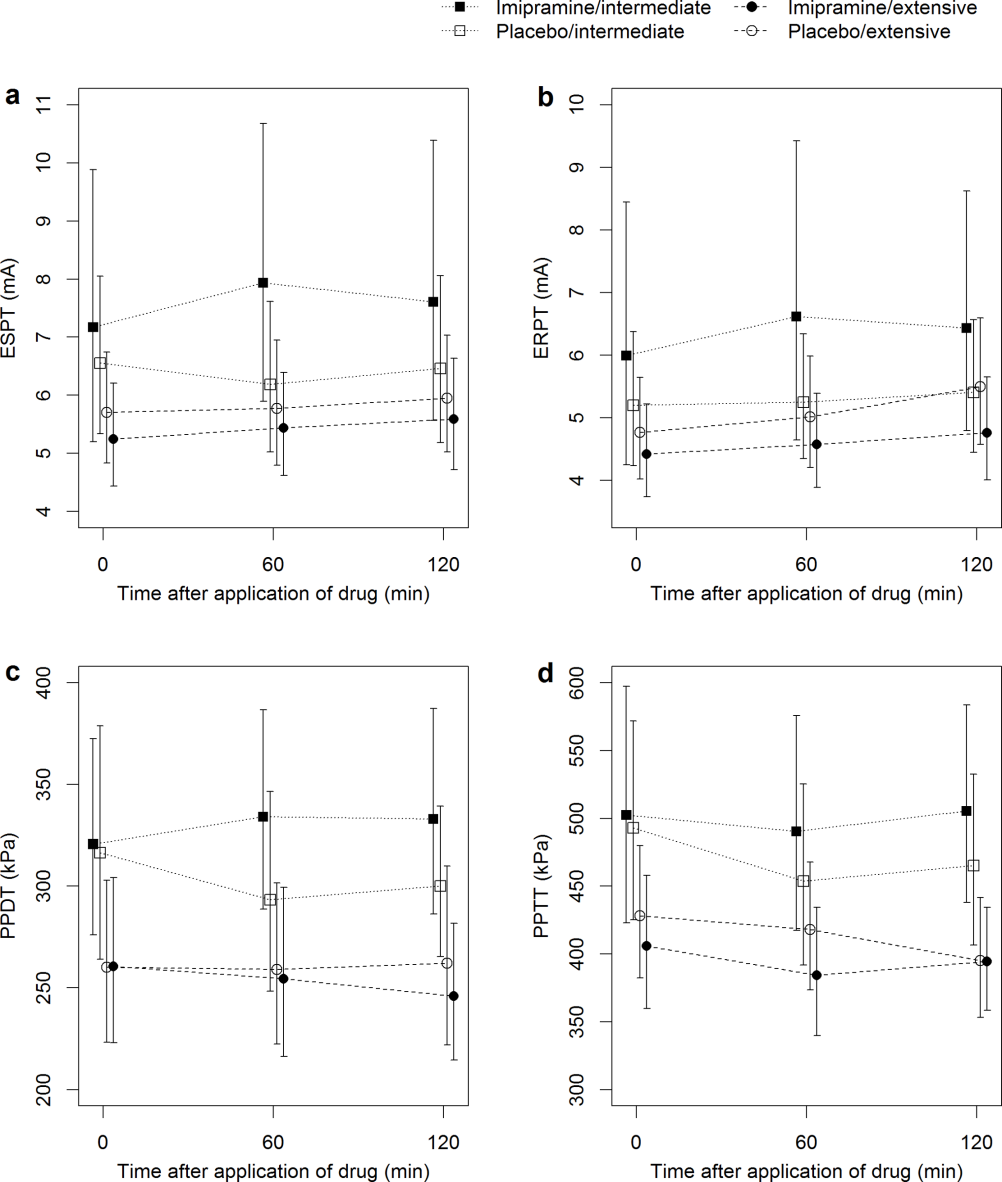
Interaction of drug effect and genotype. Effect of imipramine (filled symbols) vs. placebo (open symbols) on electrical pain detection threshold with a single (ESPT) or repeated stimulus (ERPT), on pressure pain detection (PPDT) and tolerance thresholds (PPTT) for intermediate (squares) and extensive (circles) CYP2D6 metabolizers. The interaction between imipramine effect and CYP2D6 genotype is suggested by the diverging lines within the first 60 minutes in intermediate metabolizers (squares), whereas no such divergence is seen in extensive metabolizers (circles).

After imipramine, intermediate metabolizers had a tendency towards higher PPDT after 60 and 120 minutes (1.10 (1.01 to 1.21), and 1.07 (0.97 to 1.17)), whereas extensive metabolizers showed no such tendency (interaction p = 0.054). Intermediate metabolizers had 1.07 times higher PPTT 120 minutes after imipramine compared to placebo (95%-CI 1.00 to 1.17, p = 0.047), but a dependency on CYP2D6 metabolizer status was not found (interaction p = 0.475). No significant differences between imipramine and placebo and hence no interaction between genotypes were observed in the thermal QST. After imipramine, intermediate metabolizers took longer until the cold pressor pain reached NRS 7/10 than after placebo (mean ratios 1.20 (1.02 to 1.42), and 1.17 (0.98 to 1.40) at 60 minutes (p = 0.032) and a little less so after 120 minutes (p = 0.08). An illustration of the interactions is displayed in Fig 3. Table 2 displays the detailed results for electrical, mechanical and cold pressor tests. The results of thermal QST are displayed in S1 Table.

**Table 2:** Quantitative sensory tests in intermediate and extensive metabolizers.

	Time	2D6 subtype	Marginal mean ratio (95% CI)	p value	p value for interaction
ESPT	60	intermediate metabolizer	1.20 (1.10 to 1.31)	0.000	0.007
		extensive metabolizer	1.03 (0.96 to 1.11)	0.403	
	120	intermediate metabolizer	1.15 (1.04 to 1.26)	0.006	
		extensive metabolizer	0.99 (0.90 to 1.07)	0.736	
ERPT	60	intermediate metabolizer	1.08 (0.98 to 1.18)	0.111	0.079
		extensive metabolizer	0.97 (0.89 to 1.05)	0.451	
	120	intermediate metabolizer	1.05 (0.96 to 1.16)	0.284	
		extensive metabolizer	0.95 (0.87 to 1.03)	0.223	
PPDT	60	intermediate metabolizer	1.10 (1.01 to 1.21)	0.034	0.054
		extensive metabolizer	0.99 (0.91 to 1.07)	0.722	
	120	intermediate metabolizer	1.07 (0.97 to 1.17)	0.169	
		extensive metabolizer	0.96 (0.88 to 1.04)	0.278	
PPTT	60	intermediate metabolizer	1.01 (0.94 to 1.08)	0.767	0.475
		extensive metabolizer	0.98 (0.92 to 1.04)	0.506	
	120	intermediate metabolizer	1.07 (1.00 to 1.15)	0.047	
		extensive metabolizer	1.04 (0.98 to 1.11)	0.210	
Iwsec	60	intermediate metabolizer	1.20 (1.02 to 1.42)	0.032	0.231
		extensive metabolizer	1.05 (0.91 to 1.21)	0.483	
	120	intermediate metabolizer	1.17 (0.98 to 1.40)	0.080	
		extensive metabolizer	1.03 (0.88 to 1.20)	0.722	

Effect of imipramine vs. placebo on pain detection threshold with a single (ESPT) or repeated electrical stimulus (ERPT), and on pressure pain detection (PPDT) and tolerance thresholds (PPTT), and the time until cold pain reaches 7 on NRS (Iwsec) for different CYP 2D6 genotypes (intermediate vs. extensive metabolizers). The effect is estimated on the log scale and presented as a mean ratio. A significant interaction would indicate that the effect differs between genotypes.

### Side effects

Nausea, dizziness and sedation occurred in both the imipramine and the tolterodine session.

Overall, side effects were rated very low, but nausea and dizziness occurred significantly more after imipramine than after tolterodine. After 120 min, nausea was rated 0.3 (SD 0.9) in the imipramine arm and 0.0 (0.1) in the tolterodine arm (p = 0.025). Dizziness after 120 min was rated 0.6 (1.4) after imipramine and 0.1 (0.4) after tolterodine (p = 0.002). Overall, sedation was the strongest side effect after 120 min with 1.4 (2.2) in the imipramine and 1.1 (1.8) in the tolterodine arm. However, this was not significantly different (p = 0.452). Table 3 shows the ratings of side effects for both drugs at every time point.

**Table 3:** Occurrence of nausea, dizziness and sedation after drug intake.

	Imipramine	Tolterodine	p-value
Nausea			
30 min	0.2 (0.8)	0.0 (0.2)	0.146
60 min	0.0 (0.1)	0.0 (0.1)	n/a
90 min	0.1 (0.5)	0.0 (0.2)	0.371
120 min	0.3 (0.9)	0.0 (0.1)	0.025
Dizziness			
30 min	0.5 (1.3)	0.1 (0.6)	0.024
60 min	0.4 (1.2)	0.1 (0.5)	0.119
90 min	0.5 (1.2)	0.1 (0.4)	0.021
120 min	0.6 (1.4)	0.1 (0.4)	0.002
Sedation			
30 min	1.2 (1.9)	1.3 (2.1)	0.755
60 min	1.3 (2.1)	1.5 (2.3)	0.460
90 min	1.3 (2.1)	1.3 (2.1)	0.963
120 min	1.4 (2.2)	1.1 (1.8)	0.452

Intensity of side effects nausea, dizziness and sedation, rated on a numeric rating scale between 0 (no side effect at all) and 10 (maximally imaginable side effect). Values are displayed as means (standard deviation).

## Discussion

This study failed to demonstrate an immediate analgesic effect of a single oral dose of imipramine on non-specific low-back pain. Anti-nociceptive effects as assessed by QST were only observed in intermediate metabolizers of CYP2D6, after additional subgroup analyses.

### Imipramine and low-back pain

There are two possible reasons why no significant analgesic effect of imipramine on low-back pain was detected in the present study. The first one is that a single oral dose may not be sufficient. The literature commonly states that antidepressants start to be effective only after several days [2,15]. This may be true for neuropathic pain conditions, where the benefit of antidepressants is well documented. Nevertheless, there is no convincing explanation for this delayed effect. Earlier studies have found TCAs to be effective in chronic low-back pain [4–6,16] after several weeks. However, none of these studies assessed whether an immediate analgesic effect already occurred after the first dose.

A mechanistic proof of analgesia or anti-nociception requires a correlation in time between drug intake and effect, ideally after single dose administration. Such a correlation can actually be seen with the side effects of TCAs, like sedation or dry mouth which occur soon after drug intake. When the analgesic effect of TCAs occurs with a delay, it may more likely be due to mood stabilization and improved sleep quality, leading to overall improved clinical well-being, rather than to analgesia.

The second explanation may be that imipramine is not effective in low-back pain at all. A closer look at the literature reveals that only two studies have evaluated the effect of imipramine on chronic low-back pain [4,17], and only a few more have investigated other tricyclic compounds [5,6,16]. The results are remarkably controversial. Jenkins et al. report no significant improvement after imipramine compared to placebo [17], whereas Alcoff et al. conclude that imipramine was potentially useful [4]. Several systematic reviews on tricyclic antidepressants for chronic low-back pain come to different conclusions [18–21], depending on which original articles they include. The most recent study [22] used amitriptyline and concluded that it was superior to pregabalin after 14 weeks of treatment. This was, however, an open-label trial without placebo control. The latest Cochrane review from 2008 does not at all support the use of antidepressants in chronic low-back pain [21]. The results of the present study do not imply that imipramine is ineffective in low-back pain, but suggest mechanisms of action other than analgesia.

### Imipramine and QST

The anti-nociceptive properties of imipramine have been well documented in several previous studies. In animals, these effects are nearly immediate, and can be measured 5 minutes after intravenous [23] or 1 hour after intraperitoneal administration [24,25]. In human volunteers, anti-nociceptive effects significantly superior to placebo were detected 90 minutes after oral administration [7]. The present study, however, showed no effect in the primary analyses at that time or later.

There are several possible explanations for this discrepancy. Firstly, two hours observation time may have been insufficient. But given that peak plasma concentrations of imipramine are reached between 2 and 3 hours [26], one would at least expect a trend in the last QST measurements after 2 hours. Another explanation might be the choice of QST modalities. The study of Poulsen et al. [11] showed a significant effect of imipramine on pain tolerance thresholds, but none on pain detection thresholds. This could subsequently be replicated by Enggaard et al. [10]. With the exception of PPTT (for which, notably, an anti-nociceptive trend was observed), the stimuli in the present work were not exceedingly intense and therefore, relevant effects may have been missed. More intense pain paradigms such as electrical pain tolerance, ischemic pain or a capsaicin model may be considered for future studies. A study by Wallace et al. [27] found that skin touch as well as warm and cold sensation thresholds remained unchanged by desipramine. They concluded that desipramine had no effect on acute nociception, but they had not tested any pain tolerance thresholds. The most intriguing explanation, however, why imipramine had no effect in the in the present study may be that the onset of anti-nociception depends on the speed of drug metabolism. Imipramine is partly transformed to its active metabolite desipramine by N-demethylation via CYP2C19. Both imipramine and desipramine are hydroxylated to 2-Hydroxy-imipramine and 2-Hydroxy-desipramine by CYP2D6, which are the rate-limiting steps [28]. These occur somewhat faster in extensive than in intermediate metabolizers. During the short observation period of two hours, when enteral absorption may still be in progress, plasma levels rise more slowly in extensive metabolizers because more of the active compounds are hydroxylated by CYP2D6. This may explain why only intermediate metabolizers showed anti-hyperalgesic effects at that time. The overall analysis showed no net effect on QST, because the effect in intermediate metabolizers was counterbalanced by a lack of effect in extensive metabolizers. Anti-hyperalgesic effects may therefore become evident only when genotypes are analyzed separately.

To our knowledge, none of the previous studies took different CYP2D6 genotypes into account. Rather, Poulsen et al. [11] had explicitly enrolled only extensive metabolizers of imipramine. Trends to anti-nociceptive effects in these extensive metabolizers were noted 3–6 hours after drug intake. In the present study, extensive metabolizers might as well have shown such effects, had the observation time been longer. Intermediate metabolizers, on the other hand, started to show anti-nociceptive effects already after 2 hours. One might hypothesize that both intermediate and extensive metabolizers of imipramine experience anti-nociceptive effects, but that they occur sooner in intermediate and only later in extensive metabolizers.

### Strengths and limitations

Most of the previously published work was performed on healthy volunteers using sample sizes between 10 and 20 individuals [7–11,27]. In contrast, the present study enrolled chronic pain patients, and the results are therefore more clinically relevant. Our sample is by far the largest one that examines imipramine and QST. Patients presented with homogeneous clinical findings, and were properly randomized and blinded. Given the possible delayed onset of action of TCAs, administration of a single oral dose may be debatable. However, the study aimed at detecting an analgesic effect. If this had been clinically relevant, it should have been detectable even after single dose administration. The relatively short observation time of 2 hours is a limitation of the study, as is the lack of measurements of imipramine plasma concentrations. It is known that plasma concentrations depend on metabolizer status [29], but not whether analgesic or anti-hyperalgesic effects correlate with plasma concentrations. Demonstrations of lacking drug effect despite adequate plasma concentrations would have supported our conclusions. The interaction analysis was not defined in the study protocol and has to be considered a post-hoc analysis with exploratory character. Multiple statistical testing has been performed and has to be taken into consideration when interpreting the reported p-values, since no correction for multiple comparisons was attempted.

## Conclusions

The present study revealed no analgesic effect after a single oral dose of imipramine within 2 hours in chronic low-back pain patients. It does not necessarily challenge the clinical practice of prescribing TCAs in chronic low back pain. It suggests, however, that their effects may not be the result of an analgesic process in the classical sense. Other actions, such as mood and sleep modulation, may be more important than a direct analgesic effect. Furthermore, the study suggests that imipramine may have anti-nociceptive properties that depend on CYP2D6 metabolizer status and become evident only in experimental but pain tests. This finding has not been reported before and should be taken into account when planning future studies using tricyclic antidepressants.

## Supporting information

S1 Appendix Study protocol. (DOCX)

S2 Appendix CONSORT-checklist. (PDF)

S1 Table Results of quantitative sensory tests. Effect of Imipramine vs. placebo on heat pain detection (HPDT) and tolerance thresholds (HPTT) at either arm or leg at each time point for different CYP2D6 genotypes (intermediate vs. extensive metabolizers). The temperature was limited to a maximum of 50.5°C and the treatment effect was therefore estimated by tobit regression models. Cold pain detection threshold (CPDT) was dichotomized into patients with a CPDT of 0°C (CPDT at limit) vs. patients with CPDT above 0°C. A significant interaction would indicate that the effect differs between genotypes.
(DOCX)
