# Supplementary material for: Effect of single-dose imipramine on chronic low-back and experimental pain. A randomized controlled trial
Source: PLoS One. 2018 May 9;13(5):e0195776. doi: 10.1371/journal.pone.0195776 (PMC5942791; doi:10.1371/journal.pone.0195776)
Supplement: S1 Table — Effect of Imipramine vs. placebo on heat pain detection (HPDT) and tolerance thresholds (HPTT) at either arm or leg at each time point for different CYP2D6 genotypes (intermediate vs. extensive metabolizers). The temperature was limited to a maximum of 50.5°C and the treatment effect was therefore estimated by tobit regression models. Cold pain detection threshold (CPDT) was dichotomized into patients with a CPDT of 0°C (CPDT at limit) vs. patients with CPDT above 0°C. A significant interaction would indicate that the effect differs between genotypes. (DOCX) [file pone.0195776.s003.docx]

|  | Time | 2D6 subtype | Marginal mean difference (95% CI) | p value | p value for interaction |
| --- | --- | --- | --- | --- | --- |
| HPDT (leg) | 60 | intermediate metabolizer | 1.90 (0.63 to 5.72) | 0.256 | 0.229 |
|  |  | extensive metabolizer | 0.77 (0.30 to 1.98) | 0.584 |  |
|  | 120 | intermediate metabolizer | 2.05 (0.75 to 5.63) | 0.163 | 0.937 |
|  |  | extensive metabolizer | 1.95 (0.83 to 4.56) | 0.126 |  |
| HPDT (arm) | 60 | intermediate metabolizer | 2.47 (0.36 to 16.81) | 0.354 | 0.405 |
|  |  | extensive metabolizer | 0.85 (0.18 to 4.11) | 0.843 |  |
|  | 120 | intermediate metabolizer | 1.08 (0.19 to 6.33) | 0.929 | 0.903 |
|  |  | extensive metabolizer | 0.94 (0.22 to 4.00) | 0.933 |  |
| HPTT (leg) | 60 | intermediate metabolizer | 1.72 (0.91 to 3.25) | 0.098 | 0.054 |
|  |  | extensive metabolizer | 0.77 (0.47 to 1.26) | 0.304 |  |
|  | 120 | intermediate metabolizer | 1.40 (0.62 to 3.17) | 0.421 | 0.567 |
|  |  | extensive metabolizer | 1.04 (0.57 to 1.89) | 0.903 |  |
| HPTT (arm) | 60 | intermediate metabolizer | 1.35 (0.39 to 4.65) | 0.632 | 0.999 |
|  |  | extensive metabolizer | 1.35 (0.53 to 3.44) | 0.529 |  |
|  | 120 | intermediate metabolizer | 1.18 (0.34 to 4.10) | 0.798 | 0.915 |
|  |  | extensive metabolizer | 1.08 (0.41 to 2.84) | 0.878 |  |
|  |  |  |  |  |  |
|  |  |  | Marginal odds ratio  (95% CI) |  |  |
| CPDT (leg) at limit | 60 | intermediate metabolizer | 1.00 (0.40 to 2.50) | 1.000 | 0.889 |
|  |  | extensive metabolizer | 1.08 (0.64 to 1.83) | 0.765 |  |
|  | 120 | intermediate metabolizer | 1.69 (0.53 to 5.36) | 0.372 | 0.512 |
|  |  | extensive metabolizer | 1.06 (0.55 to 2.05) | 0.863 |  |
| CPDT (arm) at limit | 60 | intermediate metabolizer | 0.62 (0.24 to 1.60) | 0.321 | 0.537 |
|  |  | extensive metabolizer | 0.90 (0.48 to 1.67) | 0.732 |  |
|  | 120 | intermediate metabolizer | 0.74 (0.36 to 1.56) | 0.434 | 0.148 |
|  |  | extensive metabolizer | 1.53 (0.93 to 2.51) | 0.091 |  |

S1 table
